# Third Trimester Serum Amino Acid Metabolism is Associated with Maternal Breast Cancer Diagnosed within 15 years of Pregnancy

**DOI:** 10.21203/rs.3.rs-3272893/v1

**Published:** 2023-08-23

**Authors:** Sami Teeny, Zachery R. Jarrell, Nickilou Y. Krigbaum, Piera M. Cirillo, Young-Mi Go, Barbara A. Cohn, Dean P. Jones

**Affiliations:** Emory University; Emory University; Public Health Institute; Public Health Institute; Emory University; Public Health Institute; Emory University

**Keywords:** Amino acids, Early onset breast cancer, Metabolomics, Pregnancy

## Abstract

A prospective metabolome-wide association study revealed widespread amino acid limitation in late pregnancy is associated with early onset breast cancer. Archival third trimester pregnancy serum samples from 172 women who subsequently were diagnosed with breast cancer within 38 years after pregnancy were compared to 351 women without breast cancer. No individual metabolite differed after false discovery rate adjustment, indicating that individual metabolites are unlikely to be useful for classification or prediction. Despite this, pathway enrichment analysis showed that amino acid pathways, including lysine, arginine, proline, aspartate, asparagine, alanine, tyrosine, tryptophan, histidine, branched-chain amino acid and urea cycle, were enriched among metabolites that differed at raw p < 0.05. Several of these pathways previously were linked to breast carcinogen exposures, including dichlorodiphenyltrichloroethane and perfluorinated alkyl substances. Network analyses showed that amino acids correlated with parity and the ratio of estriol to estrone and estradiol known risk factors for breast cancer in this cohort. Overall, amino acid associations were stronger for early onset breast cancer, defined here as occurring within the first 15 years following pregnancy. Although results must be interpreted cautiously, lower amino acid concentrations for histidine, threonine and proline, and stronger associations for tryptophan, histidine, and lysine pathways with breast cancer within 15 years, suggests that amino acid limitations during late pregnancy contribute to metabolic reprogramming that is causally related to early onset breast cancer. Environmental chemical effects on nutrient sensing could account for these effects through known oncogenic mechanisms linked to nutrient stress.

## Introduction

Breast cancer is a leading cause of early mortality and the most common cancer in woman, with over 2.26 million new cases worldwide in 2020 ^1^. In the mid-20th century, researchers reported an association of young age at first pregnancy with lifetime protection against breast cancer [1, 2]. This observation has since been qualified by consistent reports of a transient increase in risk of breast cancer after pregnancy [3]. The magnitude and interval of increased risk depend upon a woman’s age and parity at the time of her pregnancy [4]. The lifetime protection of pregnancy in the long term has been linked to pregnancy-associated changes in breast cells that increase resistance to malignant transformation [5], while the transient increase in risk has been linked to a possible promotion of an existing lesion by pregnancy hormones or to inflammation during involution of ductal tissue postpartum [4]. Currently, however, there is no consensus regarding underlying molecular mechanisms for pregnancy associations with breast cancer [6].

In prior multigenerational research on maternal environmental chemical exposures, liquid chromatography-high-resolution mass spectrometry (LC-HRMS) of selected archival serum samples in the Child Health and Development Studies (CHDS, Public Health Institute, Oakland California) were used with metabolome-wide association studies (MWAS) to find metabolic associations with concentrations of dichlorodiphenyltrichloroethane (DDT) [7] and perfluorinated alkyl substances (PFAS) [8], previously shown to influence breast cancer in daughters. Pathway enrichment analysis showed associations of these environmental carcinogens with differences in both non-essential and essential amino acid pathways. These findings have important implications for causal mechanisms in cancer because amino acids are used for protein synthesis and growth, and also have critical non-protein functions in nutrient sensing [9], inflammation [10], blood pressure regulation [11, 12], and neurotransmission [13].

To further test whether amino acid changes during pregnancy are linked to breast cancer outcomes in women, we focused on a 1960’s pregnancy cohort previously used to study associations of serum estrogens during pregnancy with subsequent breast cancer diagnosis. In that study, the ratio of estriol (E3) to the sum of estrone (E1) and estradiol (E2) in third trimester serum samples was found to be protective from subsequent breast cancer [14]. The association was stronger for cancers diagnosed within the first 15 y after pregnancy, for primiparas, and for women who were older than the median age (27 y) of the cohort at the time of pregnancy. We used a metabolomics workflow in which we first performed untargeted analyses to select top discriminatory features, followed by pathway enrichment analysis to provide an agnostic test of amino acid pathway associations with breast cancer outcome. Upon finding associations, targeted testing of amino acid metabolites and network analyses were used to clarify relationships of amino acids to prior measures of estrogens, age, parity, and interval between pregnancy and cancer diagnosis.

## Materials and Methods

### Human study population

Archival serum samples were from the Child Health and Development Studies (CHDS, Public Health Institute, Oakland, California). The CHDS recruited women residing in the Oakland, California area who were members of the Kaiser Foundation Health Plan and received obstetric care for pregnancies between 1959 and 1967 [15]. Breast cancer cases were identified by linkage to the California Cancer Registry and the California Vital Status Records for cases diagnosed through 1997 as previously described [16]. Record abstraction is based primarily on pathology reports, and case identification is considered to be > 99% complete after a 2-year lag for cancer diagnoses [16]. Cases were defined as women with incident invasive or noninvasive breast cancer diagnosed at a median age at diagnosis of 54 years (interquartile range, 13 years) with available prenatal serum. Third (T3) trimester samples of 172 women who subsequently developed breast cancer were analyzed along with samples from 351 women from the cohort who did not develop breast cancer [17]. Of women with breast cancer, 34 had cancer diagnosis within 15 years of pregnancy. The institutional review board of the Public Health Institute approved the present study, and we complied with all federal guidelines governing the use of human participants.

Characteristics of the subjects studied are provided in Table 1. Age, parity, and estrogen concentrations for estrone (E1), estradiol (E2), and estriol (E3) were as previously described [14]. In this prior study, covariate analyses showed that age and the log of the concentrations of E1, E2, and E3, as well as the log of the ratio of E3 to E1 and E2, were associated with breast cancer diagnosis by either Welch’s t-test or chi-square test.

### Chemicals

HPLC grade acetonitrile and methanol, LC-MS water and 98% formic acid were obtained from Sigma-Aldrich (St. Louis, MO). A mixture of 14 stable isotopic chemicals were used as internal standards [18], including [^13^C6]-D-glucose, [^15^N]-indole, [2-^15^N]-L-lysine dihydrochloride, [^13^C5]-L-glutamic acid, [^13^C7]-benzoic acid, [3,4-^13^C2]-cholesterol, [^15^N]-L-tyrosine, [trimethyl-^13^C3]-caffeine, [^15^N2]-uracil, [3,3-^13^C2]-cystine, [1,2-^13^C2]-palmitic acid, [^15^N,^13^C5]-L-methionine, [^15^N]-choline chloride and 2’-deoxyguanosine-15N2,^13^C10-5’-monophosphate from Cambridge Isotope Laboratories, Inc (Andover, PA).

### High-resolution metabolomics (HRM)

Serum samples were randomized into batches of 20 samples, for extraction and analysis using liquid chromatography-high resolution mass spectrometry (LC-MS) as described previously [19, 20]. Briefly, 50 μL serum sample was treated with 2:1 (v/v) with acetonitrile, and 2.5 μL internal standard of a mixture of the 14 stable isotope standards. Following mixing, extracts were incubated at 4°C for 30 min, and protein precipitates were removed by centrifugation for 10 min at 21000 × g at 4°C. Supernatants were placed in autosampler vials and maintained at 4°C in an autosampler prior to analysis. A pooled human reference material [Qstd, pooled plasma from 2 lots (Equitech-Bio, Inc, Kerrville, Texas)] was analyzed at the beginning and end of each batch of 20 samples. A second pooled human reference material (NIST SRM1950) was analyzed at the beginning and end of the study. Samples were analyzed in triplicate by liquid chromatography (LC) coupled with Q-Exactive HF mass spectrometer (Thermo Fisher). A dual chromatography using C18 and HILIC with electrospray ionization (ESI) in negative and positive ion mode, respectively, was utilized. Data collection occurred continuously throughout 5 min of chromatographic separation from *m/z* 85 to 1,275.

### HRM Data Processing and Feature Selection

Peak detection, noise removal, and alignment of retention times and features were performed using the adaptive processing for LC-MS data (apLCMS) v.6.6.8 R package [17, 19–21]. Additional filtering of low-quality samples and features and batch correction were conducted with xMSanalyzer v.1.3.2 [22]. Mass spectral features for each sample consisted of mass to charge (*m/z*), retention time (rt), and associated integrated ion intensity; interpretations are limited to features with annotation or identification as metabolites and are therefore termed metabolites. Only samples with Pearson correlation ρ > 0.7 among 3 replicates were retained. Both xMSanalyzer and apLCMS were run using R v.4.0.3. Metabolic features were filtered to retain those with median coefficient of variation (CV) among technical replicates < 70%. Intensities below the detection threshold were not retained by the extraction procedures, and detected intensities were median summarized across triplicates for statistical analyses.

### Untargeted Metabolomics Feature Statistics

Untargeted analyses were performed with procedures to balance Type 1 (false positives) and Type 2 (false negatives) statistical error due to the expectation that the population studied was not yet in an active disease state at time of blood collection and effect sizes may be small [23]. Hence, rigorous selection to minimize risk of Type 1 error poses the risk of missing important associations. Feature intensities were log2 transformed to decrease heteroscedasticity. One-way ANOVA using the limma R package was used with R v.4.0.3 to test for differential expression at raw p < 0.05, and the Benjamini-Hochberg Procedure was used to test for false discovery. Selected features at raw p < 0.05 were used with Partial Least-Squares Discriminant Analysis (PLS-DA) to identify features that best distinguished the control and breast cancer groups. PLS-DA score plots and 2-way hierarchical cluster analysis were used for visualization of separation of groups based upon selected features. Features with p < 0.05 and VIP > 1 were used for pathway enrichment analysis with mummichog version 2 (MetaboAnalyst) [24], using combined HILIC + ESI and C18 −ESI data.

### Metabolite Identification

Identities of amino acids and other selected metabolites were confirmed by accurate *m/z* match and co-elution with authentic standards [Level 1 identification by criteria of Schymanski et al] [25]. Other features were annotated with a multistage clustering algorithm, xMSannotator [26] using the HMDB database [27] at 5 ppm tolerance. For Level 1 or higher annotations, these are considered equivalent to Level 3 and in combination with mummichog pathway analyses, equivalent to Level 3 criteria of Schymanski et al. [25].

### Bioinformatics and Network Analysis

Metabolome-wide association studies (MWAS) were performed using the xmsPANDA R package (https://github.com/kuppal2/xmsPANDA). We tested all identified amino acids for associations with phenotypic and demographic and feature data using xMWAS [28]. Thresholds for inclusion in the network were |r| > 0.40 and p < 0.05. We also plot a Pearson correlation matrix of all amino acids with hierarchical clustering using the heatmap library in R [20]; to simplify the interpretation of the heat map, amino acid adducts were only included if they possessed at least one correlation with another network node where |r| > 0.4.

### Covariate Analysis

Demographics and covariate analysis results of the subjects are summarized in Table 2 and Table 3. The only measure found to significantly differ between the control and the breast cancer group was age. No correction for age was applied in later analysis, as attempting to remove the influence of age would likely mask important metabolites and increase Type 2 error.

## Results

### Metabolic features associated with subsequent breast cancer

**Figures 1:**
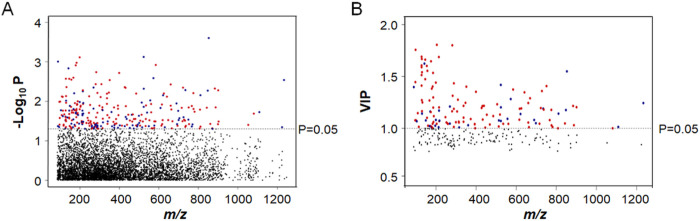
Manhattan Plots for C18 analyses. Left figure shows limma selection followed by PLS-DA selection off the limma-filtered subset (p<0.05) on the right (VIP>1.0). Red indicates a selected metabolite with a positive correlation to breast cancer diagnosis, while blue indicates a selected metabolite with a negative correlation.

Limma showed that 275 metabolites in C18 analyses differed by case status at raw p < 0.05 (Fig. 1A). No metabolites were significant after FDR correction, indicating that none is likely to be useful as an individual biomarker. A similar pattern of results was observed for 261 metabolites in HILIC analyses (data not shown). Collectively, these results show that associations of individual metabolites in pregnancy serum with subsequent breast cancer are relatively weak and unlikely to be useful as independent early predictors of breast cancer outcome. PLS-DA analysis of the C18 data showed that 136 metabolites contributed to separation, of which 33 were positively and 103 negatively associated with breast cancer (Fig. 1B). For HILIC, 128 metabolites contributed to separation, 33 positively and 95 negatively.

### Top features associated with breast cancer provide poor breast cancer classification

**Figure 2:**
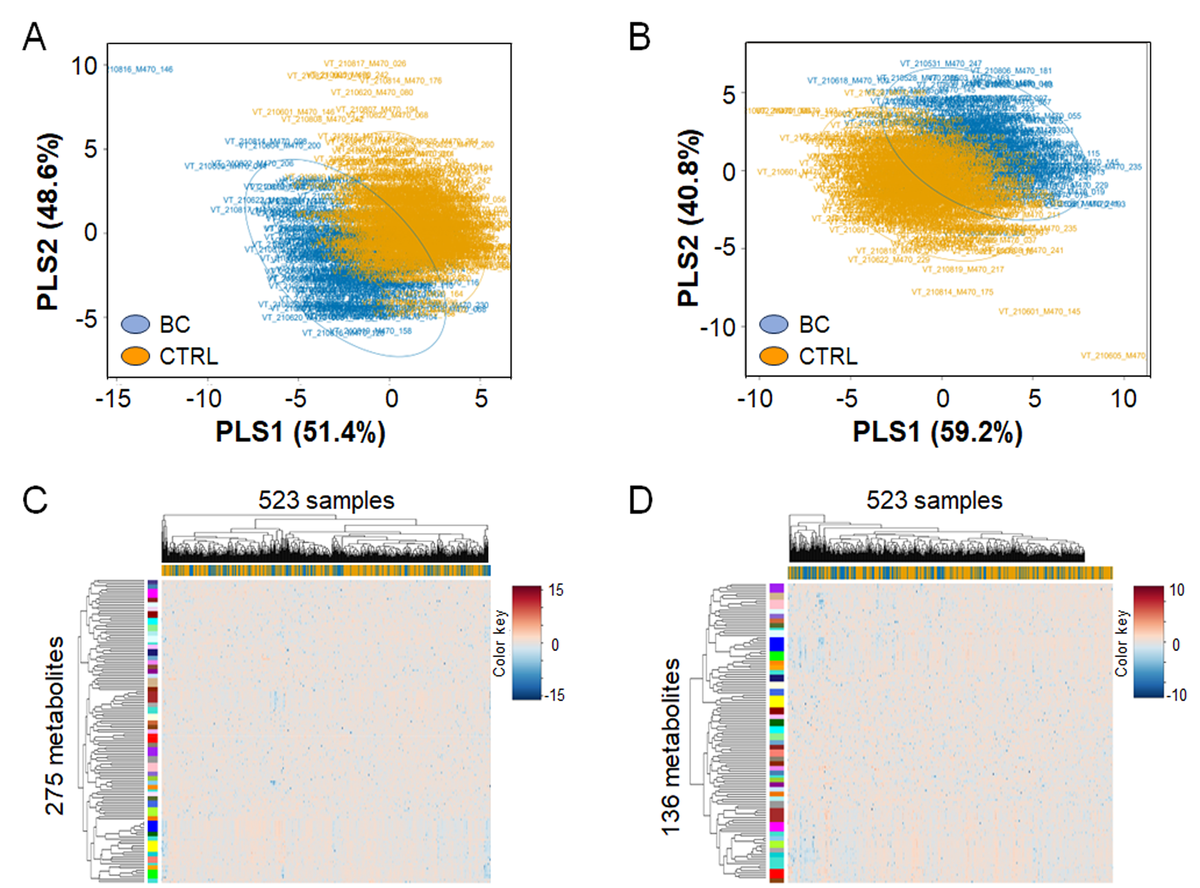
PLS-DA Score Plots and Hierarchical Cluster Analysis (HCA) of selected discriminatory metabolites for breast cancer outcome. **A.** PLS-DA for C18 metabolites. **B.** PLS-DA for HILIC metabolites. **C.** HCA for C18 metabolites. **D.** HCA for HILIC metabolites. For PLS-DA Score Plots, the blue group represents the set of metabolites positively correlated with breast cancer diagnosis, while the yellow group shows the negatively correlated metabolites. For HCA, the blue group in both figures represents the set of metabolites positively correlated with breast cancer (BC) diagnosis, while the yellow group (CTRL) shows the negatively correlated metabolites. In the HCA plots, positive correlations are shown in orange/red while negative correlations are shown in blue.

Visual examination of PLS-DA score plots for breast cancer and non-breast cancer subjects using the top selected C18 and HILIC metabolites showed partial but incomplete separation of groups (Fig. 2A,B). Hierarchical cluster analysis of these selected metabolites showed some subclusters of breast cancer cases (blue color in band associated with top dendogram) separating from non-cancer individuals (yellow color associated with top dendogram; Fig. 2C, D), which may represent metabolic subclasses of women who subsequently develop breast cancer. A short list of metabolites with VIP > 1.5 is provided in supplementary material (**Additional file 1. Table S1**) with p values and fold changes, illustrating that the metabolites with strongest associations with breast cancer had relatively low fold-changes relative to non-cancer individuals.

#### Pathway enrichment analysis of metabolites associated with subsequent breast cancer

**Figure 3:**
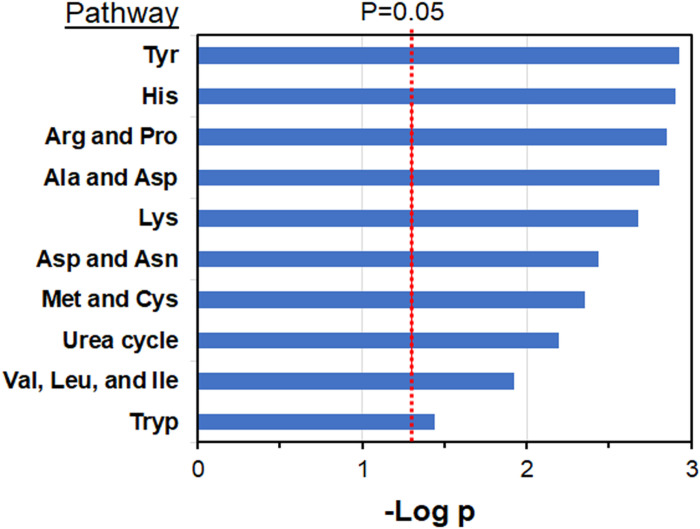
Amino acid pathways in third trimester pregnancy serum associated with subsequent breast cancer diagnosis. Metabolites differing between non-cancer and cancer cases were selected at p < 0.05 and used with permutation testing for pathway enrichment using *mummichog*. Other associated pathways are provided in supplementary material, Additional file 3, Table S3. Breast cancer cases, n = 172; non-cases, n = 351.

Pathway enrichment analysis showed that the selected metabolites were enriched in nine amino acid pathways and the urea cycle (Fig. 3). Pathways associated with breast cancer included tyrosine; histidine; arginine and proline; alanine and aspartate; aspartate and asparagine; lysine; methionine and cysteine; valine, leucine and isoleucine; and tryptophan. These pathways included amino acids and metabolites with Level 1 identification, including proline, histidine, urocanate, lysine, threonine, phenylalanine, phenylpyruvate, 5-oxoproline (pyroglutamate). Additional support for pathway identifications was obtained by Pearson correlation analyses of metabolites, which provided additional confirmation for all pathways except the aspartate and asparagine pathway and the methionine and cysteine pathway (**supplementary material, Additional file 2, Table S2**). Additional pathways associated with breast cancer are provided in **supplementary material, Additional file 3, Table S3;** these are not studied further here to retain focus on an apparent generalized association of amino acid metabolism and subsequent breast cancer risk.

### Network analysis of amino acid associations with breast cancer

**Figure 4:**
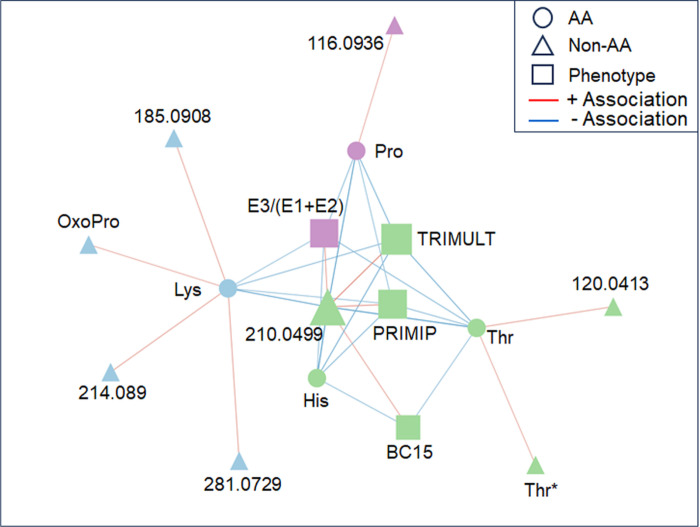
Network analysis of amino acids, phenotypic measures and other top features associated with breast cancer. Node color indicates community, node size indicates centrality, and edge color indicates direction of correlation where blue corresponds to negative correlation and red to positive correlation respectively. Nodes with unknown identity are annotated with their ionization mode and accurate mass. Community 1 centers on TRIMULT, PRIMIP, BC15, threonine, and histidine, Community 2 centers on lysine and 5-oxoproline and Community 3 centers on E3/(E1+E2) and proline. AA, amino acid; OxoPro, 5-oxoproline.

We explored community relationships of amino acids with selected phenotypic measures, age, and other metabolites that passed selection criteria. Analyses were performed with a data-dependent community detection tool, xMWAS, with an intent to identify in an untargeted manner the central communities that are relevant to amino acid metabolism and breast cancer. Phenotypic measures included breast cancer status (BC), breast cancer diagnosis within 15 y (BC15), primiparous status (PRIMIP), ≥ 3 live births (TRIMULT), and log-transformed estrogen concentrations for E1, E2, E3, and the ratio of E3/(E1 + E2). In this analysis, a PLS correlation threshold of r > 0.4 was combined with Eigenvector Centrality Scores to generate networks of communities (Fig. 4). Three communities were observed, distinguished by color, Community 1, green; Community 2, blue; and Community 3, magenta. In this analysis, a metabolite with *m/z* 210.0499 was most central. This accurate mass matches the M + 2Na^+^-H^+^ adduct of phenylalanine in positive ESI; however, in correlation analyses (**Supplementary material, Additional file 2, Table S2**), this did not correlate with other adduct forms of phenylalanine, so we consider this unidentified.

Of the phenotypic measures, TRIMULT had the highest Eigenvector Centrality Score (scale is zero to one, with one having highest centrality), followed by PRIMIP and E3/(E1 + E2). TRIMULT and PRIMIP were in the largest community, Community 1 (green), which also included BC15, two adduct forms of threonine, histidine and two unidentified metabolites. The next largest community, Community 2 (blue), contained lysine as a hub connected to an adduct form of histidine, 5-oxoproline, and two unidentified metabolites. Lysine was connected to multiple nodes in Community 1 and also to E3/(E1 + E2), one of two hubs in Community 3 (magenta). Community 3 included proline, which also had multiple edges connected to nodes in Community 1, and an unidentified metabolite. Notably, the phenotypic measures, BC15, PRIMIP, E3/E1 + E2, and TRIMULT, all had significant associations to identified amino acids, while age did not have any associations that were strong enough to be included in this network. Histidine and threonine showed strong negative correlations to BC15. All of the amino acids included in the network, including proline, lysine, threonine, and histidine, showed negative correlations to PRIMIP, TRIMULT, and E3/E1 + E2. Also worth noting from this analysis, BC15 had a relatively high Eigenvector Centrality Score despite only having correlations at this threshold with histidine, threonine and the unidentified central metabolite (*m/z* 210.0499).

### Quantified Metabolites

**Figure 5:**
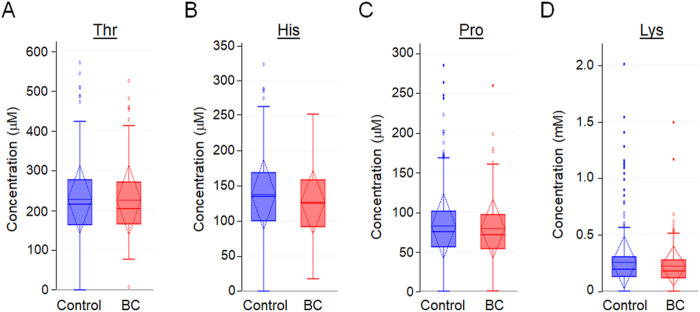
Threonine, histidine, proline and lysine, amino acids associated with central hubs in network analysis, show only small differences between breast cancer and non-breast cancer **cases.** Concentrations (mM) are represented with Box and Whisker Plots, where the dotted lines show the mean and standard deviations of the metabolites. Concentrations for breast cancer (BC) and non-breast cancer (control) groups showed higher median values in the non-cancer group for all selected amino acids.

Amino acids present in the central communities in Fig. 4, including threonine, histidine, proline and lysine were quantified to allow direct comparisons of differences between individuals who subsequently developed breast cancer and those who did not. As is evident for these amino acids (Fig. 5), even though the populations differed at p < 0.05. the populations had substantial overlap so that, as indicated above, individual amino acids have little discriminatory usefulness.

### Metabolome-wide association of breast cancers occurring within 15 y of sample collection

**Figure 6:**
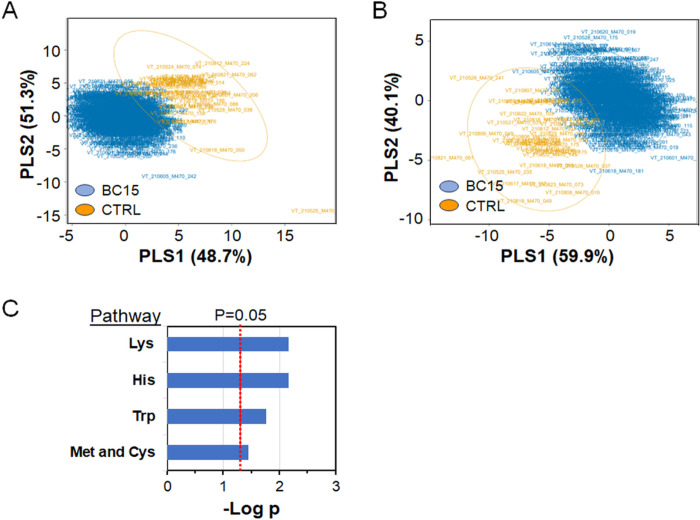
PLS-DA Score Plots of BC15 and associated pathway enrichment analysis. **A.** Comparison of BC15 (n = 34) to non-BC15 group (CTRL, n = 489) in C18 data. **B.** Comparison of BC15 (n = 34) to non-BC15 group (CTRL, n = 489) in HILIC data. **C.** Mummichog selected pathways with selected metabolites differing for BC15 compared to all others.

The Eigenvector Centrality Score for BC15 suggested important relationships for this group. The top associations with threonine, histidine and the unidentified *m/z* 210.0499, occurred without BC15 having similar associations with PRIMIP, TRIMULT, and E3/E1 + E2. These findings indicated that BC15 may have different metabolic antecedents than breast cancer diagnosed after 15 y. To investigate this, we repeated our statistical analysis using individuals with breast cancer within 15 years of pregnancy as our target cohort and the remaining subjects (BC > 15 y and non-cases) for reference. Limma analysis for C18 provided 237 selected metabolites (61 positive associations, 176 negative associations) at p < 0.05. For HILIC analysis, there were 233 selected metabolites (78 positive associations, 155 negative associations) at p < 0.05. Application of PLS-DA to these metabolites with selection criteria of VIP > 1.0 resulted in 99 selected metabolites (9 positive associations, 90 negative associations) for C18 and 113 selected metabolites (25 positive associations, 88 negative associations). The PLS-DA Score Plots for these selected metabolites (Fig. 6A,B) showed that overall separation was superior for BC15 than for all BC with comparison to non-breast cancer (Fig. 2A) or for comparison to BC cases diagnosed after 15 years (Fig. 2B). Pathway enrichment analysis of the selected metabolites further showed that this separation was associated with a smaller number of pathways (Fig. 6C), including histidine, lysine, tryptophan and methionine and cysteine.

## Discussion

The present MWAS of third trimester pregnancy serum with subsequent breast cancer outcome adds to current understanding of short term and lifelong effects of pregnancy on breast cancer risk. The most substantive generalization is that subsequent risk is increased in association with lower abundance of essential and non-essential amino acids in the third trimester serum. Thus, in addition to transient increased risk due to promotion of existing lesions by pregnancy hormones or inflammation [4], and increased resistance to malignant transformation of breast cells in the long term [5], this research shows an apparent generalized decrease in amino acids in multiple pathways related to protein metabolism.

Considerable nutrition research has focused on dietary protein needs during pregnancy to ensure maternal health as well as health of the newborn. Among the physiological, biochemical and structural changes that occur during pregnancy, glomerular filtration rate is increased, and this results in increased urinary loss of amino acids. This loss, combined with increased needs for protein synthesis and use of amino acid oxidation to support 20% of the energy needs for the growing fetus, results in an overall increase in need for maternal protein intake during late pregnancy by about 30%. Thus, the generalized effects on multiple amino acid pathways suggest that in women who subsequently develop breast cancer there is either 1) an insufficient protein supply from food consumption or absorption, 2) an excessive non-compensated urinary amino acid loss intake, 3) an increased fetal or placental demand for amino acids or 4) a combination of these factors resulting in decreased amino acid levels.

These decreased amino acid levels could contribute in a causal manner to breast cancer risk through metabolic reprogramming that promotes oncogenic mechanisms or alters efficacy of tumor suppressor or immune surveillance mechanisms. Alternatively, these differences could reflect differences in sensitivity to metabolic stress revealed by pregnancy, which also occur in individuals at risk for breast cancer, but are not in causal mechanisms. This could be a consequence of genetics, diet, microbiome, fetal demand, health conditions or other determinants. Critically, mechanistic studies will be needed to establish whether decreased serum amino acid concentrations in late pregnancy are causally related to subsequent breast cancer development.

The most central amino acids in network analyses, histidine, lysine, threonine and proline, were among top metabolites selected using limma- and PLS-DA-based selection. Each was negatively associated with breast cancer diagnosis, and pathway enrichment analysis also showed changes in histidine, lysine and proline pathways are associated with subsequent breast cancer. Furthermore, in network analyses, all four of these amino acids showed associations with pregnancy-related parameters, parity and the ratio of E3/(E1 + E2), indicating fundamental associations with pregnancy in addition to other possible pregnancy-independent determinants of amino acid levels. Thus, even though pathway enrichment analyses show associations with tryptophan, tyrosine, branched-chain amino acids, methionine and cysteine, and other non-essential amino acid pathways, the available data suggest a central role for histidine, lysine, threonine and proline.

The network analyses also showed relative strong, negative associations of histidine and threonine to breast cancer in the first 15 years after pregnancy. Thus, if these levels are mechanistically linked to subsequent breast cancer, low levels of these amino acids may be especially important drivers of breast cancer development. Histidine was among the top 20 metabolites associated with breast cancer outcome for the whole dataset, as judged by p-value and VIP score. In addition to protein synthesis, histidine is decarboxylated to histamine and deaminated to urocanic acid. Urocanic acid was one of the metabolites negatively associated with breast cancer outcome, and although no prior associations with breast cancer are available, urocanic acid has been associated with protection against melanoma. Histamine was not among the top metabolites associated with breast cancer outcome but is an immune modulator with receptors that have been implicated in regulation of breast cancer growth [29]. Consistent with the general concept that low histidine could contribute to oncogenesis, histidine supplementation makes cancer cells more susceptible to the effects of the chemotherapy [30]. Additionally, the tumor suppressor carnosine also contains histidine, and elevated blood histidine concentrations that are observed in cancer could be related to carnosine metabolism or decreased carnosine uptake by cancer cells [31]. Threonine was also significantly lower in blood concentration in association with breast cancer, with a relatively strong network connection to breast cancer within 15 years of pregnancy. Prior research has emphasized an important role for threonine as a precursor for glycine to support folate-dependent methylation reactions controlling the epigenome [32], and targeted research will be needed to evaluate potential contribution of decreased threonine to early onset breast cancer.

Decreased proline is also of interest concerning subsequent breast cancer risk because this can activate a stress response to reprogram non-essential amino acid metabolism as occurs in cancer cells. Myc regulates expression of key enzymes in proline metabolism, and prior research shows that cancer cells reprogram proline metabolism in conjunction with the Warburg effect to support tumor cell growth demands [33]. Depending on cancer type, genes promoting proline catabolism behave as either tumor suppressors or oncogenes, while expression of proline synthesis genes correlate to worse outcomes in cancer [34]. Rewiring of metabolism due to low proline (https://www.nature.com/articles/s41420-020-00341-8) could therefore be an important contributor to breast cancer development because of its important role in cellular adaptability.

The implications of these findings are limited by factors inherent to the study of breast cancer and pregnancy. The incredibly dynamic environment of a pregnant individual’s serum introduces a layer of complexity which could reduce consistency of metabolic signals related to subsequent cancer development. This is important because the individuals studied may not be in an active breast cancer state for many years, suggesting that effect sizes should be small and unlikely to be detected using conservative statistical methods. The pathway and network analyses employed provide greater sensitivity for detection; however, as discussed above, the meaning of associations of metabolic clusters remains unclear and will require independent validation and mechanistic follow-up. Finally, the centrality of amino acids in all of metabolism creates a challenge to interpretation because the patterns could be due to generalized differences in protein demand or turnover that are only indirectly connected to subsequent cancer risk.

In conclusion, pathway enrichment and network analysis of untargeted metabolomics data reveal differences in amino acid metabolism in the third trimester of pregnancy that are associated with subsequent breast cancer diagnosis, especially within the first 15 years after pregnancy. These differences are associated with parity and the ratio of E3 to E1 and E2, which have been previously established to modify breast cancer risk [14]. Prior mechanistic studies support the interpretation that low amino acid levels could contribute to metabolic reprogramming that is mechanistically linked to subsequent breast cancer development.

## Figures and Tables

**Table 1:** Study population characteristics. Comparison between breast cancer (BC) and control (CTRL) groups.

Characteristic	CTRL (n = 351)	Cases (n = 172)	BC within 15 y (n = 34)
**Age at blood sample, y**	26 (8)	30 (10)	35 (7.5)
**Parity at blood sampling**	**%**	**%**	**%**
0	38	30	26
1–3	19	47	50
≥3	43	23	24
**Race**	**%**	**%**	**%**
White	73	70	62
African American	18	16	21
Hispanic	2	2	3
Asian	5	9	9
Mixed	2	3	6

**Table 2:** TContinuous Demographics. Mean, median, and standard deviation are shown for the control group (CTL) and the breast cancer group (BC) across four continuous measures. Welch’s t-test was used to compute a p-value indicating that only age differs significantly between the breast cancer and control groups.

Measure	BC, Mean ± SD	CTRL, Mean ± SD	BC, Median	CTRL, Median	Welch’s p-value
Age	30.66 ± 6.48	27.12 ± 5.75	30.00	26.00	3.37E-09
logE1	5.01 ± 1.04	5.04 ± 1.09	5.06	5.15	7.66E-01
logE2	5.80 ± 0.84	5.90 ± 0.9	5.82	5.94	2.09E-01
logE3	4.87 ± 0.57	4.91 ± 0.66	4.89	4.94	4.48E-01
logE3/E1 + E2	−1.67 ± 0.65	−1.70 ± 0.64	−1.70	−1.69	5.73E-01

**Table 3:** Categorical demographics. Counts per category per experimental group are shown for three binary categories. The Chi-square test was used to calculate a p-value indicating that none of the categorical groups differ significantly between the breast cancer (BC) and control (CTRL) groups.

Measure	BC		CTRL		Chi-Square p-value
	Yes	No	Yes	No	
White Non-Hispanic	121	51	255	96	6.55E-01
PRIMIP (Primiparous)	52	120	133	218	1.04E-01
TRIMULT (> 3 Live Births)	39	133	67	284	3.99E-01

## Data Availability

The data are available within the article, supplementary information, or available from the authors upon request.

## Abbreviations

BC: breast cancer
BC15: breast cancer occurring within the first 15 years following pregnancy
CTRL: control
DDT: dichlorodiphenyltrichloroethane
E1: estrone
E2: estradiol
E3: estriol
FDR: false discovery rate
HRM: high-resolution metabolomics
HRMS: high-resolution mass spectrometry
LC-HRMS: liquid chromatography-high resolution mass spectrometry
MWAS: metabolome-wide association study
*m/z*: mass to charge
PFAS: perfluorinated alkyl substances
RT: retention time
T2: second trimester
T3: third trimester
